# Exposure of humans to the zoonotic nematode *Dirofilaria immitis* in Northern Portugal

**DOI:** 10.1017/S0950268819001687

**Published:** 2019-09-30

**Authors:** A. P. Fontes-Sousa, A. C. Silvestre-Ferreira, E. Carretón, J. Esteves-Guimarães, C. Maia-Rocha, P. Oliveira, L. Lobo, R. Morchón, F. Araújo, F. Simón, J. A. Montoya-Alonso

**Affiliations:** 1 Laboratório de Farmacologia e Neurobiologia, Centro de Investiga Farmacológica e Inovação Medicamentosa (MedInUP), Instituto de Ciências Biomédicas Abel Salazar, Universidade do Porto (ICBAS-UP), Porto, Portugal; 2 Animal and Veterinary Research Centre (CECAV), University of Trás-os-Montes and Alto Douro, Quinta de Prados, 5000-801, Vila Real, Portugal; 3 Department of Veterinary Sciences, University of Trás-os-Montes and Alto Douro, Quinta de Prados, 5000-801, Vila Real, Portugal; 4 Research Institute of Biomedical and Health Sciences (IUIBS), University of Las Palmas de Gran Canaria, 35001 Las Palmas de Gran Canaria, Spain; 5 Clínica Veterinária de Ovar, Ovar, Portugal; 6 Serviço de Imuno-hemoterapia, Centro Hospitalar Universitário São João, EPE, Porto, Portugal; 7 EPIUnit/Instituto de Ciências Biomédicas, Abel Salazar, ISPUP, Universidade do Porto, Porto, Portugal; 8 Hospital Veterinário do Porto, Travessa Silva Porto 174, 4250-475 Porto, Portugal; 9 Faculdade de Medicina Veterinária, Universidade Lusófona de Humanidades e Tecnologias, Campo Grande, 376, 1749-024 Lisboa, Portugal; 10 Center for the Study of Animal Sciences, CECA-ICETA, University of Porto, Porto, Portugal; 11 Laboratory of Parasitology, Faculty of Pharmacy, Institute of Biomedical Research of Salamanca (IBSAL) and University of Salamanca, Salamanca, Spain; 12 Faculdade de Medicina da Universidade do Porto, Porto, Portugal

**Keywords:** *Dirofilaria immitis*, human, Portugal, seroepidemiologic study, *Wolbachia pipientis*

## Abstract

Dirofilariosis caused by *Dirofilaria immitis* (heartworm) is a zoonosis, considered an endemic disease of dogs and cats in several countries of Western Europe, including Portugal. This study assesses the levels of *D. immitis* exposure in humans from Northern Portugal, to which end, 668 inhabitants of several districts belonging to two different climate areas (Csa: Bragança, Vila Real and Csb: Aveiro, Braga, Porto, Viseu) were tested for anti-*D. immitis* and anti-Wolbachia surface proteins (WSP) antibodies. The overall prevalence of seropositivity to both anti-*D. immitis* and WSP antibodies was 6.1%, which demonstrated the risk of infection with *D. immitis* in humans living in Northern Portugal. This study, carried out in a Western European country, contributes to the characterisation of the risk of infection with *D. immitis* among human population in this region of the continent. From a One Health point of view, the results of the current work also support the close relationship between dogs and people as a risk factor for human infection

Human pulmonary dirofilariosis is an emerging zoonotic disease caused by *Dirofilaria immitis*. Humans are accidentally infected in endemic areas, where dogs act as reservoirs of the parasite and the climate conditions favour the proliferation of the mosquito vectors [1]. *D. immitis* is considered a public health concern because of its zoonotic potential; in humans, the pre-adult parasite stages form coin-shaped cysts in the branches of the pulmonary arteries, which are generally asymptomatic. These cysts are detected by imaging techniques and are often mistaken for lung tumours. Biopsy is considered the definitive diagnostic method, although its invasive nature could be a limitation; on the other hand, in biopsy only late immature and adults removed before the degeneration process elicited by the inflammatory response of the host can be reliably identified, so polymerase chain reaction (PCR) could be a valuable tool in these cases. In turn, serology techniques, currently available only as ‘in house’ tests, could help to diagnose the nature of the pulmonary cysts, by detecting the presence of antibodies against *D. immitis* and its symbiotic bacteria *Wolbachia*; thus, human pulmonary dirofilariosis should be considered in the differential diagnosis of compatible lesions, especially in endemic areas [2, 3].

*D. immitis* causes heartworm disease in dogs and cats, which is endemic in Southern European countries, including Greece, Italy, Spain and Portugal. In these countries, the prevalence has increased in some regions [4, 5], while others with consolidated prophylactic programs have reported decreased prevalence [6]. In humans, in 2012, there were reported 33 cases of pulmonary dirofilariosis in Europe, although, in endemic regions, the frequencies of human infections are probably higher than reported in the literature because pulmonary nodules may be unnoticed or be easily misdiagnosed [2].

Regions with high temperature and humidity favour mosquito proliferation and the presence of canine and human dirofilariosis [2]; moreover, the disease is expanding to colder areas in Eastern and Northern regions of Europe [7–9], as demonstrated by recent studies [2, 8–16]. The limits of this expansion are fuzzy but could be higher than estimated given that cases have been diagnosed in dogs (although imported) from Nordic countries such as Finland [15].

In Portugal, there is evidence of the presence of canine *D. immitis* infection in almost all regions of the country [17–20]. Also, there is an increasing number of feline *D. immitis* infections in regions from Central, Northern and Southern Portugal [2, 18, 21]. According to the Köppen-Geiger climate classification, Northern Portugal has a warm temperate climate with dry summers (type Cs), divided into two subtypes: Csa, with hot summers with the average temperature in the warmest month above 22 °C and Csb, with warm summers with the average temperature in the hottest month below or equal to 22 °C and with 4 months or more with the average temperatures above 10 °C [22].

To our knowledge, two previous reported cases of pulmonary nodules by *D. immitis* in Portugal demonstrated the risk of infection among the Portuguese population [23]; however, no seroepidemiological study to assess this risk has been previously published. In the present study, we demonstrate, for the first time, the exposure to *D. immitis* of people living in Northern Portugal.

For this cross-sectional study, 668 human serum samples from two local hospitals (Centro Hospitalar S. João, Porto, Portugal and Centro Hospitalar de Trás-os-Montes e Alto Douro, Vila Real, Portugal) were analysed between July 2013 and November 2014. Inclusion criteria included people living in the area of interest of the study, who had not travelled outside the country in the last 6 months and agreed to participate. The samples were randomly selected among those who fulfilled the inclusion criteria.

Of the included samples, 333 (49.85%) were from males and 335 (50.15%) from females, ranging from 2 to 95 years (median 49 years, 36–67 interquartile range (IQR)). The number of samples by age group was 168 for ⩽35 years (25.10%), 193 for 36–50 years (28.90%), 133 for 51–65 years (19.90%) and 174 for ⩾66 years (26.0%). Serum samples were collected from people living in six districts of Northern Portugal, with Csa (Bragança, Vila Real) and Csb climates (Aveiro, Braga, Porto, Viseu). The pattern of distribution by age and gender was representative of the population living in Northern Portugal according to the 2011 census data [24]. The distribution of samples by gender, age and district of residence is shown in Table 1.

**Table 1.** Seroprevalence of human dirofilariasis in Northern Portugal, as defined by seropositivity for antibodies against both *D. immitis* and WSP

	Male				Female				Total			
	*n*	Positive			*n*	Positive			*n*	Positive		
		*n*	%	95% CI		*n*	%	95% CI		*n*	%	95% CI
	333	21	6.3	4.2–9.5	335	20	6.0	3.9–9.0	668	41	6.1	4.6–8.2
Age (years)												
⩽35	74	5	6.8	2.3–14.9	94	7	7.4	3.7–14.6	168	12	7.1	4.1–12.1
36–50	108	7	6.5	3.2–12.8	85	2	2.4	1.0–8.2	193	9	4.7	2.5–8.6
51–65	71	2	2.8	1.0–9.7	62	4	6.5	2.5–15.5	133	6	4.5	2.1–9.5
⩾66	80	7	8.8	4.3–17.0	94	7	7.4	3.7–14.6	174	14	8.0	4.9–13.1
Study sites												
*Csa*	137	11	8.0	4.5–13.8	172	10	5.8	3.2–10.4	309	21	6.8	4.5–10.2
Bragança	9	1	11.1	2.0–43.5	17	0	0.0	0.0–18.4	26	1	3.9	1.0–18.9
Vila Real	128	10	7.8	4.3–13.8	155	10	6.5	3.5–11.5	283	20	7.1	4.6–10.7
*Csb*	196	10	5.1	2.8–9.1	163	10	6.1	3.4–10.9	359	20	5.6	3.6–8.5
Porto	166	9	5.4	2.9–10.0	135	7	5.2	2.5–10.3	301	16	5.3	3.3–8.5
Viseu	20	0	0.0	0.0–16.1	24	3	12.5	4.3–31.0	44	3	6.8	2.4–18.2
Braga	4	0	0.0	0.0–49.0	2	0	0.0	0.0–65.8	6	0	0.0	0.0–39.0
Aveiro	6	1	16.7	3.0–56.4	2	0	0.0	0.0–65.8	8	1	12.5	2.2–47.1

This research conforms to the Declaration of Helsinki and was approved by the ethics committee of the hospitals included in the study. The confidentiality of the information of the patients was always maintained and all of them provided written consent to participate in the study.

To estimate the seroprevalence of human *D. immits* exposure, samples were analysed by serological techniques for anti-*D. immitis* and anti-*Wolbachia* antibody detection, as previously described [25], with some modifications. In brief, 96-well microplates were coated with 0.8 µg of an extract of *D. immitis* somatic antigen and *Wolbachia* surface protein (WSP). Samples were prepared at 1:100 for anti-*D. immitis* serum antibodies and 1:40 for anti-WSP antibody detection. The secondary antibody (anti-human IgG peroxidase-conjugated; Merck, Germany) was used at 1:5000. An Easy Reader (Bio-Rad Laboratories, USA) was used to measure the optical densities at 492 nm. Cut-off points of *D. immitis* (0.8) and WSP (0.5) were obtained as the arithmetic mean optical density ± 3 standard deviations of 20 samples from clinically healthy blood donors living in a *D. immitis*-free area. People were considered seropositive when anti-*D. immitis* and anti-WSP antibodies presented jointly [4, 18, 26].

Data were analysed using the SPSS^®^ 25 software for Windows (SPSS Inc./IBM, Chicago, Illinois, USA). Descriptive analysis of the considered variables was carried out considering the proportions of the qualitative variables. The *χ*^2^ or Fisher's exact tests were used to compare percentages of positives among categories of the same independent variables and also the total prevalence of *D. immitis*. The individual data were analysed as a dependent variable by the logistic regression model, using *D. immitis* status as the outcome. In all cases, the significance level was established at *P* < 0.05.

Of the studied samples, 41 were positive to anti-*D. immitis* and anti-WSP antibodies. Therefore, the seroprevalence was 6.1%. No statistically significant differences were observed between males and females (6.3% and 6.0%, respectively) and there were seropositive inhabitants from 19 to 88 years old (median age 50.0 years, 33.0–75.5 IQR). Seroprevalences were 7.1% (12/156) in inhabitants ⩽35 years, 4.7% (9/184) from 36 to 50 years, 4.5% (6/127) from 51 to 65 years and 8.0% (14/160) in those ⩾66 years (*P* > 0.05). Considering the climate areas, a prevalence of 6.8% in the Csa and 5.6% in the Csb climate zones was observed, with no significant differences between them (*P* > 0.05). The seroprevalences by district were 12.5% (Aveiro), 7.1% (Vila Real), 6.8% (Viseu), 5.3% (Porto), 3.9% (Bragança) and 0% (Braga). The baseline characteristics of the study population and its reactivity to *D. immitis* and WSP are summarised in Table 1. A map showing the study areas and the distribution of the positive subjects is shown in Figure 1.

**Fig. 1.**
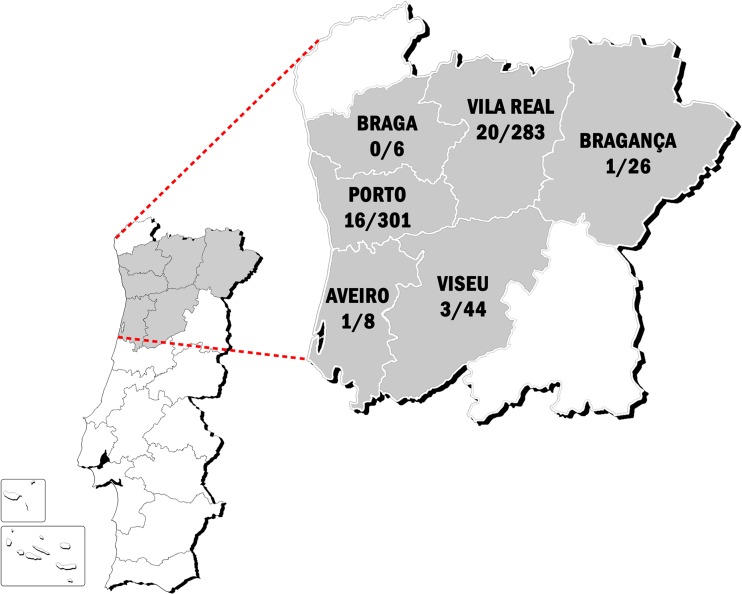
Map of Portugal showing the areas included in the current study and the distribution of the positive subjects *vs*. the total subjects evaluated per area.

Finally, the univariate logistic regression analysis identified no statistically significant association between the evaluated independent variables (gender, age, climate area of residence) and positive serology to *D. immitis* antigens and WSP.

Heartworm infection prevalence is increasing in Western and Mediterranean European countries, while it is currently also being reported in Northern and Eastern areas of the continent. This is due to a number of factors, including climate change, the emergence of new species of mosquitoes able to transmit parasites, a greater transport of reservoir dogs from endemic areas and human modification of environment, such as irrigating lands for farming, which contributes to the spread of the dirofilariosis by allowing development and activity of the vector [7].

*D. immitis* infection is a zoonosis and a vector-borne disease. Humans are exposed to this disease through the bite of infected mosquitoes and, as it is an emerging disease, it is not surprising that increasing of human dirofilariosis cases are being reported in endemic countries [2]. Zoonotic *D. Immitis*, as well as seropositive inhabitants, have been reported in Western Europe [25–30] and Eastern Europe [13, 31–34]. A similar pattern has been observed with *D. repens*, being more frequently reported in recent years [13, 35, 36].

Several studies confirmed Portugal as an endemic country for animal heartworm infection [37]. Regarding the area evaluated in the current work, a seropositivity of 15% in cats and between 2.1% and 27.3% in dogs has been observed [18]. Similarly to as reported in other endemic areas, infected dogs seem to constitute a risk of transmission to human [38]. However, in areas where the seroprevalence of human heartworm infection was high (e.g. Porto, Vila Real and Viseu), no canine heartworm infection has been reported to date [18]. Thus, further studies are needed to update the data of canine heartworm infection prevalence in these districts; however, being Portugal a small country, it facilitates travelling between endemic and non-endemic areas. On the other hand, the highest prevalence was found in Aveiro, where the presence of *D. immitis* has been demonstrated in dogs (6.8%) and cats (18.7%) [18], suggesting that the inhabitants have a high risk of infection. Similar findings have been reported in the nearest country – Spain –in La Rioja [27] and the Canary Islands [39]. There is a lack of seroepidemiological studies in other European regions, such as Eastern countries, although new studies are demonstrating the presence of seropositive inhabitants in Romania, Moldova and Serbia [13, 33]. Regarding transmission, presence of competent vectors as well as *D. immitis* infections has been described in mosquitoes in other areas of Portugal [40, 41] and neighbouring areas of Spain [42, 43].

The overall results presented the highest seroprevalence in inhabitants under the age of 35 years old (7.1%) and above the age of 66 years old (8.0%), similar to previous studies in other endemic areas [26, 39].

Although pulmonary dirofilariosis has been described in Portugal [23], awareness of human infections among Portuguese physicians is poor [44]. This reinforces the importance of the current study to raise awareness of the disease among the medical community. Importantly, physicians should be aware of this infection and should include pulmonary dirofilariasis in the differential diagnosis of patients presenting pulmonary nodules. Moreover, due to the increasing presence of canine heartworm infection, awareness campaigns should be carried out among veterinary clinicians and pet owners to promote the chemoprophylaxis of the disease [2, 38].

Some limitations of this study should be considered, namely the limited number of samples obtained in some districts (e.g. Aveiro and Braga) and the evaluation of only a few independent variables (e.g. age, gender and area of residence). The influence of other variables on the outcome would have been important to consider (e.g. spending time outdoors during the mosquito season, travelling to endemic areas, owning dogs and presence or absence of *D. immitis* infection in them).

In conclusion, this study described, for the first time, the seroreactivity to *D. immitis* and WSP in the human population living in several districts of Northern Portugal. The presence of dogs infected by *D. immitis* is a potential threat to public health [13, 25, 26]. So, from the point of view of the One Health concept, it is necessary to build cooperation amongst physicians and veterinarians in the surveillance and control of this emerging zoonotic disease. Moreover, further studies are needed to understand the possible interactions between human and animal heartworm infection to guarantee its prevention and management.
